# Treadmill exercise alleviates Alzheimer’s disease pathologies in APP/PS1 mice through modulation of microglial glucose metabolic reprogramming

**DOI:** 10.3389/fnagi.2025.1734837

**Published:** 2025-12-19

**Authors:** Fei Liang, Feng Sun, Cuijun Guo, Huacong Zhong

**Affiliations:** 1 College of Physical Education and Health, Gannan Normal University, Ganzhou, China; 2 Department of Recruitment and Employment, Gannan Normal University, Ganzhou, China

**Keywords:** Alzheimer’s disease, glycolysis, microglia, oxidative phosphorylation, treadmill exercise

## Abstract

**Objective:**

Our preliminary studies have demonstrated that exercise counteracts Alzheimer’s disease (AD) by mitigating microglia-mediated neuroinflammation and enhancing microglial Aβ clearance. However, the underlying mechanism remains unclear. Given the crucial role of glucose metabolic reprogramming in regulating microglial functions, this study investigated the effects of treadmill exercise on microglial glucose metabolism and associated AD pathologies.

**Materials and methods:**

Three-month-old male APP/PS1 transgenic mice were randomly assigned to a sedentary group (AD-SED) or an exercise group (AD-EXE). Age- and sex-matched C57BL/6 mice served as the wild-type control group (WT-SED). The AD-EXE group underwent a 3-month treadmill exercise intervention. Following the intervention, we assessed spatial learning and memory using the Morris water maze test, measured neuroinflammation and Aβ levels via Western blot and ELISA, and analyzed microglial glucose metabolism using LC-MS/MS targeted metabolomics and Seahorse assays.

**Results:**

APP/PS1 mice exhibited longer escape latencies during place navigation trial and fewer platform crossings during the spatial probe trial; these deficits were partially reversed by treadmill exercise. Furthermore, the exercise intervention significantly reduced hippocampal Aβ levels and suppressed neuroinflammation. Notably, microglia from 6-month-old APP/PS1 mice showed significant impairments in both glycolysis and oxidative phosphorylation (OXPHOS), with a metabolic profile primarily reliant on glycolysis. Treadmill exercise enhanced both glycolysis and OXPHOS, and shifted the metabolic phenotype from glycolytic-dominant toward oxidative phosphorylation, and restored metabolic homeostasis.

**Conclusion:**

Treadmill exercise promotes microglial glucose metabolic remodeling, which attenuates neuroinflammation and Aβ pathology, and restores spatial learning and memory deficits in APP/PS1 mice.

## 1 Introduction

Alzheimer’s disease (AD) is a chronic neurodegenerative disorder of the central nervous system, characterized by a progressive decline in multiple cognitive domains, including memory, executive function, attention, and visuospatial abilities (Bondi et al., 2017). With the acceleration of global aging, the number of individuals living with AD and related dementia is expected to rise from 55 million in 2019 to 139 million in 2050, and the related annual costs is predicted to rise from $1.3 trillion in 2019 to $2.8 trillion by 2030 (Clare and Jeon, 2024), posing a severe challenge to the global public health system. Despite comprehensive and in-depth research into AD pathogenesis over the past few decades, clinical treatments that can effectively delay or reverse disease progression remain lacking. In this context, investigating non-pharmacological interventions strategies has emerged as a key focus in AD research.

Physical exercise, a non-pharmacological intervention characterized by high safety and minimal adverse effects, has been demonstrated to confer multiple beneficial effects on AD. Epidemiological studies indicate that exercise effectively reduces the risk of AD (Lopez-Ortiz et al., 2023; Zhang et al., 2023) and delays cognitive decline in AD patients (Zhang et al., 2022; Yuan et al., 2024). Animal studies provide further compelling evidence that exercise improves AD-related cognitive impairment (Da Costa Daniele et al., 2020; de Andrade Santos et al., 2024) and pathological hallmarks, including decreased *β*-amyloid (Aβ) accumulation (Vasconcelos-Filho et al., 2021), reduced tau hyperphosphorylation(Gutierre et al., 2025), enhanced neurogenesis and synaptic plasticity (Lourenco et al., 2019), and attenuated neuroinflammation (Wang et al., 2023). Microglia have been clearly identified as contributors to AD pathogenesis by mediating neuroinflammation and Aβ clearance. Our previous studies found that exercise not only inhibited microglial inflammatory activation (Liang et al., 2020), but also enhanced microglia-mediated Aβ clearance (Liang et al., 2022). However, the precise mechanisms underlying these exercise-induced benefits require further elucidation.

In recent years, a growing number of studies have indicated that glucose metabolism is a core mechanism regulating the functional switch of microglia between inflammatory and phagocytic states (Ghosh et al., 2018; Lepiarz-Raba et al., 2023). However, controversy remains regarding how AD alters microglial glucose metabolism. Some studies reported that AD microglia exhibit enhanced glycolysis and increased expression of glycolytic enzymes (Holland et al., 2018; McIntosh et al., 2019; Pan et al., 2019), while others found reduced activity of key enzymes in both glycolysis and oxidative phosphorylation (OXPHOS) (Zhong et al., 2024). Despite these inconsistencies, the collective findings suggest that abnormal glucose metabolic reprogramming occurs in AD microglia. Nevertheless, conclusive research evidence is still lacking on whether exercise can ameliorate AD-induced dysregulation of microglial glucose metabolism.

Building on this foundation, the present study used LC–MS/MS-based targeted metabolomics and Seahorse assays to elucidate the effects of treadmill exercise on microglial glucose metabolism in APP/PS1 transgenic mice. Concurrently, the effects of treadmill exercise on Aβ accumulation, neuroinflammation, as well as spatial learning and memory abilities were also examined. These findings will provide new evidence and mechanistic insights into the benefits of exercise in AD.

## 2 Materials and methods

### 2.1 Animals

Male APPswe/PS1dE9 (APP/PS1) transgenic mice and age-matched wild-type C57BL/6 mice were obtained from Huachuang Xinnuo (Jiangsu, China). All animals were housed in standard cages under controlled conditions (temperature 22–24 °C, humidity 50–60%, and a 12-h light/dark cycle) with ad libitum access to food and water. At 3 months of age, APP/PS1 mice were randomly assigned to either a sedentary group (AD-SED, *n* = 40) or an exercise group (AD-EXE, *n* = 40). Age-matched wild-type C57BL/6 mice served as the control group (WT-SED, *n* = 40). All experimental procedures were performed in accordance with the Guidelines for the Care and Use of Laboratory Animals (Ministry of Health, People’s Republic of China) and were approved by the Animal Use and Ethics Committee of Gannan Normal University (Approval No. gnnu2024-0624).

### 2.2 Exercise protocol

Mice in the exercise group underwent a three-month treadmill running intervention, following a protocol adapted from previous studies (Zhang et al., 2019; Liang et al., 2022). The intervention consisted of a familiarization phase followed by a formal training phase. The 6-day familiarization phase involved running at incremental speeds of 5 m/min, 8 m/min, and 12 m/min for 2 days at each speed, for 15 min per day. The subsequent formal training phase lasted 12 weeks, with sessions conducted 5 days per week for 45 min daily. Each session began with 5 min at 5 m/min, followed by 5 min at 8 m/min, 30 min at 12 m/min, and a final 5-min period at 5 m/min. All training sessions were conducted between 18:00 and 20:30. Mice in the WT and AD control groups were placed on a stationary treadmill for the same duration to control for environmental stimulation.

### 2.3 Animal allocation and sample distribution

After the exercise intervention, animals were assigned to behavioral, biochemical, and metabolic analyses. Specifically, a subset of 12 animals per group (*n* = 12) were assigned to conduct the Morris Water Maze test, and one animal in AD-EXE group was excluded due to its persistent floating behavior without active swimming in the visible platform trial. Six animals per group (*n* = 6) were assigned to perform Biochemical analyses (e.g., Elisa, Western blotting). The remaining animals per group were assigned to obtain microglia samples (WT-SED and AD-SED: *n* = 34, AD-EXE: *n* = 33). Six samples per group (*n* = 6) were used for Seahorse assays. For metabolomics analyses, in order to meet the minimum sample size requirement, every 6–7 samples per group were pooled, yielding 4 pooled samples (*n* = 4).

### 2.4 Morris water maze

The Morris water maze test was conducted over 7 days to assess spatial learning and memory. On day 1, a visible platform trial was performed with the platform raised 1 cm above the water surface. The average swimming speed was recorded to exclude potential confounding effects of visual or motor impairments on subsequent results. From days 2 to 6, the place navigation trial was conducted. Mice were introduced into the pool from four different quadrant entry points and given 60 s to locate a submerged platform (1 cm below the water surface). Any mouse that failed to find the platform within 60 s was manually guided to it and remained there for 10 s. Escape latency was recorded to evaluate spatial learning ability. On day 7, the spatial probe trial was performed by removing the platform and allowing each mouse to swim freely for 60 s. Swimming trajectory, number of platform crossings, and time spent in the platform quadrant were recorded. All data were automatically collected and analyzed using a Morris water maze video tracking system (Noldus, Netherlands). Throughout the testing period, consistent environmental conditions (lighting, temperature, and noise) were maintained for all animals to minimize external influences.

### 2.5 Protein sample preparation

Soluble and insoluble hippocampal protein fractions were prepared according to an established protocol (Velazquez et al., 2016). Briefly, six mice per group were anesthetized by intraperitoneal injection of 10% chloral hydrate (3.5 mL/kg body weight) and no typical symptoms of peritonitis were appeared until the sample preparation. Hippocampal tissues were rapidly dissected on ice and homogenized in 10 volumes of RIPA lysis buffer containing protease and phosphatase inhibitors. The homogenate was centrifuged at 100,000 × g for 1 h at 4 °C to obtain the soluble fraction (supernatant). The resulting pellet was resuspended in 70% formic acid and centrifuged again under identical conditions to collect the insoluble fraction (supernatant). Protein concentrations were normalized to 1 mg/mL using a BCA assay kit. The soluble fraction was used for Western blot and ELISA detection of soluble Aβ, while the insoluble fraction was reserved for ELISA detection of insoluble Aβ.

### 2.6 ELISA

Soluble and insoluble Aβ40 and Aβ42 levels were quantified using commercial ELISA kits (Aβ40: MEXN-H0951; Aβ42: MEXN-H0921; Meixuan). Following the manufacturer’s instructions, equal amounts of soluble or insoluble samples were added to a 96-well microplate. After sequential incubation, washing, color development, and termination steps, the optical density was measured at 450 nm using a microplate reader.

### 2.7 Western blot analysis

Western blot analysis was performed using standard procedures, including gel preparation, sample loading, electrophoresis, membrane transfer, antibody incubation, and chemiluminescent detection. The following primary antibodies were used: NLRP3 (19771-1-AP, Proteintech), ASC (10500-1-AP, Proteintech), caspase-1 p20 (341,030, Zenbio), IL-1β (A20527, Abclonal), and GAPDH (D190090, BBI).

### 2.8 Flow cytometric sorting of microglia

Following deep anesthesia, the remaining mice from each group were transcardially perfused with pre-cooled PBS. The hippocampus was rapidly dissected, and single-cell suspensions were prepared through sequential mechanical dissociation, enzymatic digestion, filtration, centrifugation, and washing steps. Cells were resuspended in staining buffer and incubated with an Fc receptor blocker for 10 min at 4 °C. Subsequently, cells were stained with CD45-FITC (E-AB-F1136C, Elabscience) and CD11b-PECY7 (E-AB-F1018HC, Elabscience) antibodies for 30 min at 4 °C in the dark. After washing and filtration, cells were resuspended in FACS buffer and sorted using a BD FACSAria III flow cytometer. The collected microglia were prepared for subsequent LC–MS/MS targeted metabolomics and Seahorse assays.

### 2.9 Metabolite extraction and LC–MS/MS targeted metabolomics

For each cell sample (10^6^ cells), 1,000 μL of pre-cooled 80% methanol aqueous solution was added. Samples were vortex-mixed, sonicated in an ice bath for 20 min, and incubated at −20 °C for 1 h. The mixtures were centrifuged at 16,000 × g for 20 min at 4 °C. The supernatant was evaporated using a high-speed vacuum concentrator. The dried samples were reconstituted in pre-cooled 50% methanol aqueous solution and centrifuged at 20,000 g 4 °C for 15 min. The supernatant was collected for LC–MS/MS analysis. Chromatographic separation was performed using a Shimadzu Nexera X2 LC-30 AD system. Mass spectrometric detection was conducted on a QTRAP 6500 + instrument (AB SCIEX) in positive and negative ion modes with multiple reaction monitoring (MRM) for metabolite detection. MultiQuant software was used to extract chromatographic peak areas and retention times.

### 2.10 Seahorse assays

The Cell Mitochondrial Stress Test and Glycolysis Rate Test were performed using a Seahorse XF96 Analyzer (Agilent, Santa Clara, US) following established protocols (Holland et al., 2018; Mela et al., 2020). Microglia were seeded into Seahorse XF culture plates at a density of 9 × 10^3^ cells per well and cultured overnight prior to the assays. For the mitochondrial stress test, the oxygen consumption rate (OCR) was measured under basal conditions and following the sequential injection of oligomycin (1.5 μmol/L, #495455, Sigma-Aldrich, UK), FCCP (0.5 μmol/L, #C2920, Sigma-Aldrich, UK), and a mixture of antimycin A and rotenone (0.5 μmol/L, #A8674, Sigma-Aldrich, UK). For the glycolysis rate test, the extracellular acidification rate (ECAR) was measured under basal conditions and after sequential injections of oligomycin (1.5 μmol/L) and 2-deoxy-D-glucose (50 mmol/L, #D8375, Sigma-Aldrich, UK). Data were recorded at 10-min intervals and analyzed using Seahorse Wave software.

### 2.11 Statistical analysis

Statistical analysis and visualization were performed using GraphPad Prism 9.3.0. All data are presented as mean ± standard error of the mean (SEM). Data from the place navigation trial were analyzed using two-way repeated measures analysis of variance (ANOVA) with training day and group as factors, followed by Tukey’s post-hoc test. For other datasets, normality and homogeneity of variance were first assessed. Data meeting both assumptions were analyzed by one-way ANOVA followed by Tukey’s test. Data with normal distribution but heterogeneous variance were analyzed using Welch’s ANOVA followed by Dunnett’s T3 test. Data not normally distributed were analyzed with the Kruskal-Wallis test followed by Dunn’s post-hoc test. A *p*-value of less than 0.05 was considered statistically significant.

## 3 Results

### 3.1 Treadmill exercise improves spatial learning and memory in APP/PS1 mice

**Figure 1:**
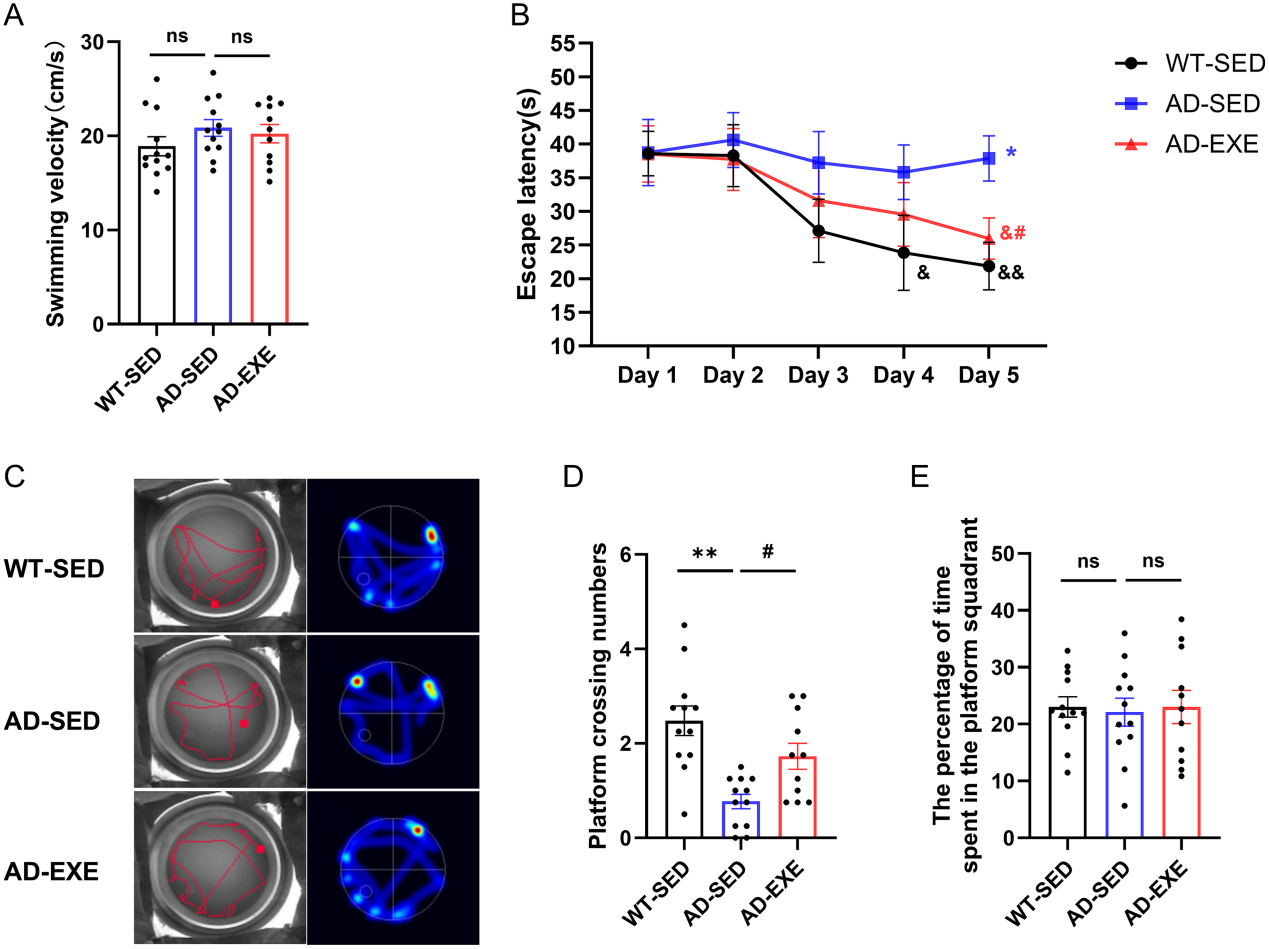
Treadmill exercise improves spatial learning and memory in APP/PS1 mice. **(A)** Average swimming speed in the visible platform trial. **(B)** Escape latency during the place navigation trial. **(C)** Representative swimming trajectories and heat maps from the probe trial. **(D)** Number of platform crossings. **(E)** Percentage of time spent in the target quadrant. &*p* < 0.05, &&*p* < 0.01 vs. day 1 within the same group; *p < 0.05, **p < 0.01 vs. the WT-SED group within the same day; #*p* < 0.05 vs. the AD-SED group within the same day.

Impaired spatial learning and memory is one of the most prominent behavioral performance of AD. To evaluate the effects of treadmill exercise on these cognitive deficits, the classic Morris Water Maze test were employed (Figure 1). The visible platform trial was conducted firstly as a critical baseline assessment, and the results showed that there was no significant difference in the average swimming speed among the three groups (*p* > 0.05, Figure 1A), eliminating the potential influence of vision and motor function on subsequent results in place navigation trial and spatial probe trial.

Subsequently, the place navigation trial was conducted and the escape latency was analyzed to evaluate the spatial learning ability (Figure 1B). Two-way repeated measures ANOVA revealed a significant main effect of training day (*F* = 10.8, *p* < 0.0001), but no significant main effect of group (*F* = 1.265, *p* = 0.296) or interaction effect between training day and group (*F* = 1.839, *p* = 0.091). The result is highly consistent with the biological logic of spatial learning, which argues that with the increase of training day, mice gradually establish memory of the platform’s spatial location, resulting in progressive reductions in escape latency. To further elucidate the spatial learning differences, post-hoc analyses were performed. Within-group comparisons indicated that compared to their respective Day 1 performance, the escape latency of the WT-SED mice was significantly shortened on Day 4 (*p* = 0.0448) and continued to shorten on Day 5 (*p* = 0.0036), while AD-EXE mice only showed a significant shorten on Day 5 (*p* = 0.0202). Between-group comparisons on the same day revealed that on Day 5, AD-SED mice exhibited longer escape latency than WT-SED (*p* = 0.0102), while the escape latency in AD-EXE mice was significantly shortened compared to AD-SED mice (*p* = 0.0475). The results indicate that 6-month-old APP/PS1 mice exhibit impaired spatial learning ability, which can be partially restored following a three-month treadmill exercise intervention.

Then, the spatial probe trial was conducted to evaluate the spatial memory ability, with the representative swimming trajectories and heat maps depicted in Figure 1C. Analysis of platform crossings (Figure 1D) demonstrated that AD-SED mice had significantly fewer platform crossings than both WT-SED (*p* < 0.0001) and AD-EXE (*p* = 0.0334) mice. However, no significant differences were observed in the percentage of time spent in the target quadrant among the three groups (*p* > 0.05, Figure 1E). These results indicate that spatial memory were impaired in 6-month-old APP/PS1 mice, and that treadmill exercise ameliorated this deficit.

Collectively, the results from Morris Water Maze indicate that 6-month-old APP/PS1 mice exhibit deficits in spatial learning and memory abilities, while the three-month treadmill exercise effectively improves these cognitive deficits.

### 3.2 Treadmill exercise reduces hippocampal Aβ accumulation and chronic neuroinflammation in APP/PS1 mice

**Figure 2:**
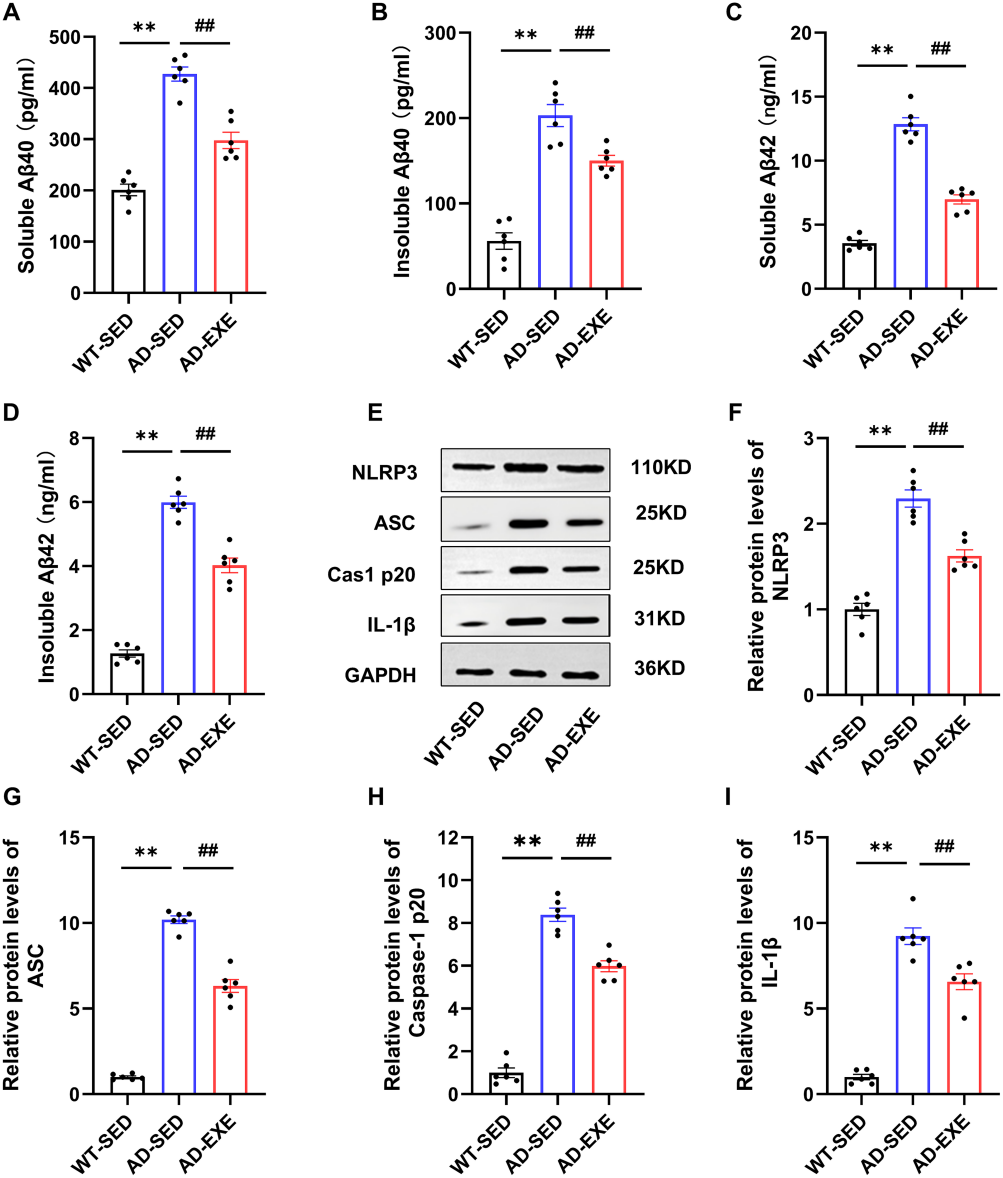
Treadmill exercise reduces hippocampal Aβ accumulation and neuroinflammation in APP/PS1 mice. **(A)** Soluble Aβ40 levels. **(B)** Insoluble Aβ40 levels. **(C)** Soluble Aβ42 levels. **(D)** Insoluble Aβ42 levels. **(E)** Representative western blot bands. **(F)** NLRP3 protein expression. **(G)** ASC protein expression. **(H)** Cleaved caspase-1 p20 protein expression. **(I)** IL-1β protein expression. **p < 0.01 vs. the WT-SED group; #*p* < 0.05, ##*p* < 0.01 vs. the AD-SED group.

Aβ accumulation is a core pathological hallmark of AD and contributes to cognitive deficits through exerting neurotoxic effects, including disrupting synaptic function, triggering neuroinflammation, etc. To examine whether treadmill exercise modulates this core pathology, ELISA was used to quantify soluble and insoluble Aβ40 and Aβ42 levels in the hippocampus (Figures 2A–D). AD-SED mice exhibited significantly elevated hippocampal levels of soluble Aβ40 (*p* < 0.0001, Figure 2A), insoluble Aβ40 (*p* < 0.0001, Figure 2B), soluble Aβ42 (*p* < 0.0001, Figure 2C), and insoluble Aβ42 (*p* < 0.0001, Figure 2D) compared with the WT-SED group, indicating a widespread Aβ accumulation. Following exercise intervention, hippocampal levels of soluble Aβ40 (*p* < 0.0001, Figure 2A), insoluble Aβ40 (*p* = 0.0052, Figure 2B), soluble Aβ42 (*p* < 0.0001, Figure 2C), and insoluble Aβ42 (*p* < 0.0001, Figure 2D) in AD-EXE mice were all significantly decreased compared with the AD-SED mice. These results indicate that treadmill exercise significantly reduces hippocampal Aβ accumulation, a key contributor that may underlie the observed cognitive improvements.

In addition to Aβ accumulation, chronic neuroinflammation is also a key pathological driver of AD progression. A line of evidence has confirmed that microglial NLRP3 inflammasome activation is a central mediator in this process, which triggers the cleavage of pro-caspase-1 into mature p20 and subsequently promoting the production of interleukin-1β (IL-1β) (Chen et al., 2023). To examine whether treadmill exercise regulates this inflammatory pathway, western blotting was used to evaluate the expression of NLRP3 inflammasome-associated proteins in the hippocampus(Figure 2E). Results showed that AD-SED mice displayed increased activation of NLRP3 inflammasome pathway compared with WT-SED mice, with significantly increased protein levels of NLRP3 (*p* < 0.0001, Figure 2F), ASC (*p* < 0.0001, Figure 2G), caspase-1 p20 (*p* < 0.0001, Figure 2H), and IL-1β (*p* < 0.0001, Figure 2I) in the hippocampus. Following treadmill exercise, AD-EXE mice showed significant reduction in protein levels of NLRP3 (*p* < 0.0001, Figure 2F), ASC (*p* < 0.0001, Figure 2G), caspase-1 p20 (*p* < 0.0001, Figure 2H), and IL-1β (*p* = 0.0003, Figure 2I) compared with AD-SED mice. These findings indicate that treadmill exercise effectively mitigates NLRP3 inflammasome-triggered neuroinflammation in APP/PS1 mice.

### 3.3 Treadmill exercise restores microglial glucose metabolism in APP/PS1 mice

**Figure 3:**
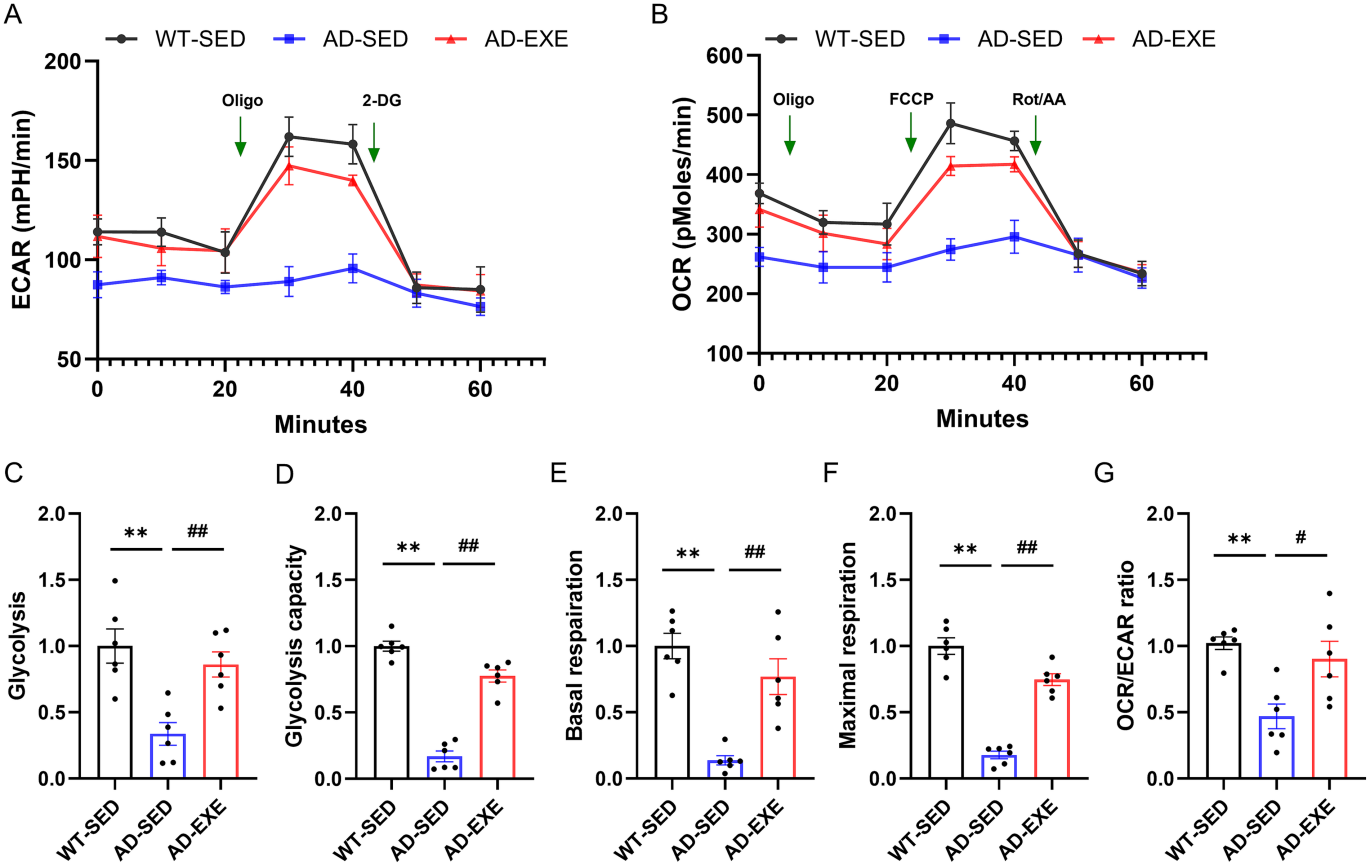
Treadmill exercise upregulates microglial glycolysis and OXPHOS in APP/PS1 mice. **(A)** ECAR measured under basal conditions and following sequential addition of Oligo and 2-DG. **(B)** OCR measured under basal conditions and following sequential addition of Oligo, FCCP, and Rot/AA. **(C)** Glycolysis. **(D)** Glycolytic Capacity. **(E)** Basal Respiration. **(F)** Maximal Respiration. **(G)** OCR/ECAR Ratio. **p < 0.01 vs. the WT-SED group; #*p* < 0.05, ##*p* < 0.01 vs. the AD-SED group.

Emerging evidence highlights that microglial glucose metabolic reprogramming from oxidative phosphorylation to glycolysis leads to their functional impairments, manifested as reduced Aβ clearance and increased inflammatory activation (McIntosh et al., 2019; Cheng et al., 2021). Both of these factors contribute to increased Aβ accumulation and chronic neuroinflammation. Given the critical role of microglial glucose metabolism and the observed exercise-induced reduction in Aβ accumulation and chronic neuroinflammation, we further examined whether treadmill exercise modulates microglial glucose metabolism. To address this, Seahorse assays were performed to measure ECAR and OCR, which indicative of glycolysis and OXPHOS, respectively. ECAR and OCR profiles was generated and presented in Figures 3A,B. Compared with WT-SED microglia, AD-SED microglia exhibited significant reductions in glycolysis (*p* = 0.0007, Figure 3C), glycolytic capacity (*p* < 0.0001, Figure 3D), basal respiration (*p* < 0.0001, Figure 3E), and maximal respiration (*p* < 0.0001, Figure 3F), indicating the impairments in both glycolysis and OXPHOS. Moreover, the OCR/ECAR ratio was also significantly decreased in AD-SED microglia compared with WT-SED microglia (*p* = 0.0169, Figure 3G), indicating a shift toward a less efficient glycolysis-dominant metabolic phenotype. Following treadmill exercise, AD-EXE microglia showed significant increases in glycolysis (*p* = 0.0095, Figure 3C), glycolytic capacity (*p* < 0.0001, Figure 3D), basal respiration (*p* = 0.001, Figure 3E), and maximal respiration (*p* < 0.0001, Figure 3F) compared with AD-SED microglia, with a concurrent increase in the OCR/ECAR ratio (*p* = 0.0439, Figure 3G). These results demonstrate that treadmill exercise restores both glycolytic and OXPHOS in microglia, while balances their contribution ratio.

**Figure 4:**
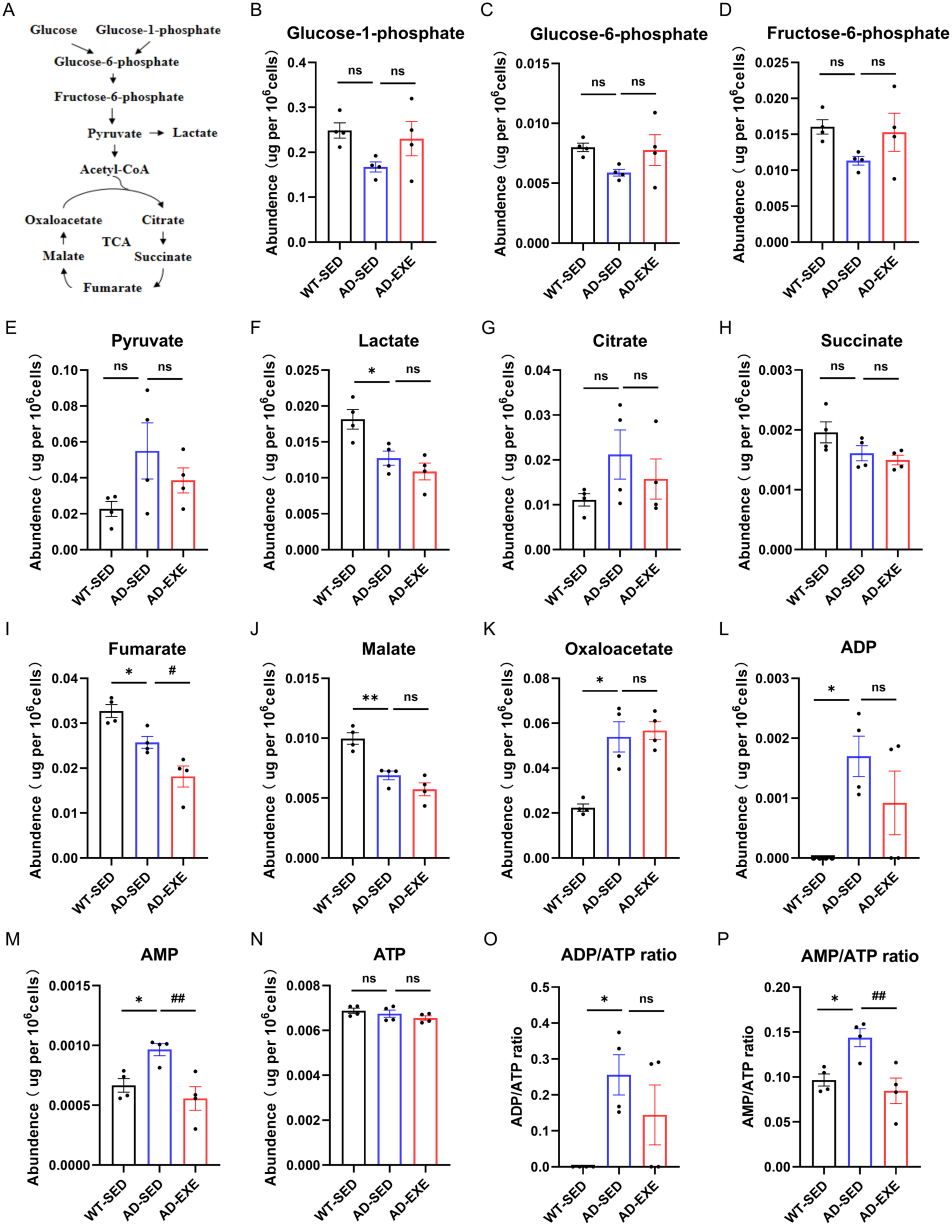
Treadmill exercise alters microglial metabolite abundance in APP/PS1 mice. **(A)** Simplified schematic. **(B)** Glucose-1-phosphate. **(C)** Glucose-6-phosphate. **(D)** Fructose-6-phosphate. **(E)** Pyruvate. **(F)** Lactate. **(G)** Citrate. **(H)** Succinate. **(I)** Fumarate. **(J)** Malate. **(K)** Oxaloacetate. **(L)**ADP. **(M)**AMP. **(N)** ATP. **(O)** ADP/ATP ratio. **(P)** AMP/ATP ratio. *p < 0.05, **p < 0.01 vs. the WT-SED group; #*p* < 0.05, ##*p* < 0.01 vs. the AD-SED group.

To validate these metabolic findings observed in Seahorse assays, LC–MS/MS targeted metabolomics was further used to quantify key glucose metabolites abundance in microglia (Figure 4). These results showed that compared with WT-SED microglia, AD-SED microglia exhibited significantly decreased levels of lactate (a glycolytic end product, *p* = 0.0246, Figure 4F), fumarate (a TCA cycle intermediate, *p* = 0.0489, Figure 4I), and malate (another TCA cycle intermediate, *p* = 0.0031, Figure 4J). The results corroborated the impairment of glycolysis and OXPHOS observed in the Seahorse assays. Moreover, AD-SED microglia also exhibited increased oxaloacetate (*p* = 0.0464, Figure 4K), ADP (*p* = 0.036, Figure 4L), AMP (*p* = 0.0401, Figure 4M), ADP/ATP ratio (*p* = 0.036, Figure 4O) and AMP/ATP ratio (*p* = 0.0306, Figure 4P), indicating a potential energy deficit. Following treadmill exercise, AD-EXE microglia showed significantly decreased fumarate (*p* = 0.0336, Figure 4I), AMP (*p* = 0.0078, Figure 4M) and AMP/ATP ratio (*p* = 0.0091, Figure 4P) compared with AD-SED microglia, indicating partial normalization of metabolite perturbations.

Collectively, these metabolic findings demonstrate that microglia from 6-month-old APP/PS1 mice exhibit impairments in both glycolysis and OXPHOS, with a metabolic profile predominantly reliant on glycolysis. Treadmill exercise partially corrects AD-induced metabolic abnormalities by enhancing both glycolysis and OXPHOS while increasing the relative contribution of OXPHOS to energy production.

## 4 Discussion

This study demonstrates that AD microglia exhibit abnormal glucose metabolic reprogramming, characterized by concurrent impairments in both glycolysis and OXPHOS, with glycolysis serving as the predominant metabolic pathway. This metabolic dysregulation was accompanied by increased hippocampal Aβ accumulation, enhanced neuroinflammation, and impaired spatial learning and memory. Following a three-month treadmill exercise intervention, both glycolytic and OXPHOS functions in microglia were enhanced, with an increased relative contribution of OXPHOS to energy production. Consequently, hippocampal Aβ accumulation and neuroinflammation were reduced, and spatial learning and memory abilities were improved.

Glucose serves as the primary energy source for microglia, the resident immune cells of the brain. Previous studies have shown that microglia primarily rely on OXPHOS for ATP production under normal physiological conditions but can rapidly shift toward glycolysis following acute lipopolysaccharide (LPS) or Aβ exposure (Baik et al., 2019; Sabogal-Guaqueta et al., 2023). To characterize the metabolic status of microglia in 6-month-old APP/PS1 mice and evaluate the effects of treadmill exercise, we assessed microglial glycolytic and OXPHOS functions using Seahorse assays and quantified relevant metabolites via LC–MS/MS targeted metabolomics. Results showed that microglia from APP/PS1 mice exhibited significantly reduced glycolysis, glycolytic capacity, and lactate levels. Concurrently, key OXPHOS indicators, including basal and maximal respiration, were markedly decreased, along with significant reductions in tricarboxylic acid (TCA) cycle metabolites fumarate and malate. These findings indicate that AD microglia display co-occurring impairments in both glycolysis and OXPHOS. The significantly decreased OCR/ECAR ratio further indicated a metabolic shift toward glycolysis as the dominant energy-producing pathway. This metabolic dysfunction coincided with elevated ADP, AMP and their respective ATP ratios in microglia. Consistent with these results, Zhong et al. (2024) reported markedly reduced activity of microglial hexokinase (HK, a key glycolytic enzyme) and pyruvate dehydrogenase (PDH, a key OXPHOS pathway enzyme) in 10-month-old APP/PS1 mice. In contrast, studies using advanced-stage AD models have reported enhanced glycolysis and increased expression of glycolytic enzymes in microglia (Holland et al., 2018; McIntosh et al., 2019; Pan et al., 2019). These discrepancies may be attributed to differences in the pathological stages of AD models and variations in methodological approaches.

Notably, following the three-month treadmill exercise intervention, AD mice exhibited significant increases in microglial glycolysis and glycolytic capacity, along with marked upregulation of basal and maximal respiration. These findings demonstrate that treadmill exercise concurrently enhances both glycolysis and OXPHOS in AD microglia. More importantly, the significantly elevated OCR/ECAR ratio indicates that the imbalance between glycolysis and OXPHOS observed in AD microglia was restored following the intervention. To our knowledge, this study provides the first evidence that exercise synergistically regulates both glycolysis and OXPHOS in AD microglia, offering novel cellular-level functional insights into how exercise ameliorates AD-related energy metabolism disorders.

Additionally, exercised microglia showed significant reductions in AMP levels and the AMP/ATP ratio, which may serve as upstream signals that negatively regulate AMP-activated protein kinase (AMPK) activation (Gowans et al., 2013), ultimately driving microglial metabolic remodeling (Trefts and Shaw, 2021; Muraleedharan and Dasgupta, 2022). Skeletal muscle, as a major endocrine organ activated by exercise, contributes substantially to this regulatory network through the secretion of myokines. A line of evidence has demonstrated that exercise intervention is able to induce the production and release of muscle-derived myokines, such as brain-derived neurotrophic factor (BDNF), Irisin and Cathepsin B into the circulation (Moon et al., 2016; Lourenco et al., 2019). Notably, these myokines can traverse the blood–brain barrier into the brain parenchyma and synergistically activate the BDNF–TrkB pathway. The activation of this pathway not only contribute to the increased glucose uptake and glycolysis through increasing the expression of HIF-1α (Zhang et al., 2020), but also promotes mitochondrial biogenesis through activating PGC-1α (Agrawal et al., 2014). In addition to myokines, exercise-induced elevation in hepatic ketone metabolite *β*-hydroxybutyrate may provide another critical regulatory axis for microglial glucose metabolism. Specially, exercise effectively enhances the production of β-hydroxybutyrate (Sleiman et al., 2016), which can be transported to the brain and exert dual regulatory effects on microglial metabolism. It not only upregulates the expression of BDNF (Sleiman et al., 2016), but also preserves mitochondrial function by inhibiting mitochondrial calcium uniporter (MCU)(Jin et al., 2023). The synergistic effects of these two pathways may jointly drive microglial metabolism reprogramming. Meanwhile, exercise may optimize microglial glucose metabolism through a dual lactate supply mechanism. On one hand, exercise enhances the glycolytic activity of skeletal muscle and elevates blood lactate levels, the circulating lactate then enter into the brain parenchyma (Park et al., 2021). On the other hand, exercise stimulates astrocyte to augment glycogenolysis and results in enhanced lactate release into the extracellular space (Matsui et al., 2017). These extracellular lactate can be internalized into microglia and serve as an alternative energy substrate to reduce their reliance on endogenous ATP consumption, thereby lowering the intracellular AMP/ATP ratio. Moreover, the internalized lactate can also act as an upstream signaling molecule that activates PGC-1*α* and promotes mitochondrial biogenesis (Park et al., 2021; Longhitano et al., 2023), which may further contribute to microglial OXPHOS enhancement. Additionally, exercise also increases cerebral blood flow and enhances cerebral oxygenation (Lu et al., 2020; Moeini et al., 2020), the optimized oxygen supplement may further augment microglial OXPHOS.

Furthermore, we also found that the three-month treadmill exercise markedly reduced hippocampal Aβ levels, alleviated NLRP3-mediated chronic neuroinflammation, and improved behavioral performance in Morris Water Maze. These results align with previous findings (Da Costa Daniele et al., 2020; Vasconcelos-Filho et al., 2021; Yang et al., 2022) and further corroborate the beneficial effects of exercise on AD pathology and associated cognitive deficits. Such benefits may be partly attributed to the restoration of microglial glucose metabolism. Mounting evidence highlights the association between microglial glucose metabolic phenotype and AD pathologies, and indicates that microglial metabolic reprogramming from OXPHOS to glycolysis serves as a key driver of AD progression (Bennett and Liddelow, 2019). This metabolic reprogramming directly impairs microglial phagocytic functions, leading to inefficient Aβ engulfment and subsequent pathological Aβ accumulation (McIntosh et al., 2019; Pan et al., 2019; Chen et al., 2025). Concurrently, it also primes microglial inflammatory activation and increases the production of pro-inflammatory cytokines (Cheng et al., 2021). In contrast, exercise-induced restoration of microglial glucose metabolism may create a functional rescue that targets both Aβ pathology and neuroinflammation. As demonstrated in our previous study, microglia-mediated Aβ clearance was markedly enhanced following a three-month treadmill exercise intervention (Liang et al., 2022). Simultaneously, microglial M1 polarization and the expression of pro-inflammatory cytokines (e.g., IL-1β, TNF-α) were decreased (Zhang et al., 2019). Although exercise-induced microglial metabolic remodeling may not be the sole contributor to the amelioration of AD pathologies, these findings support the plausibility of microglial metabolic remodeling as a critical intermediary mechanism.

There are some methodological limitations in the present study. One notable limitation is the absence of a wildtype exercise group, which may preclude definitive clarification of whether the observed benefits are specific to the AD pathological context or represent general responses to exercise intervention. Another notable limitation is the use of overnight ex vivo culture of FACS-isolated microglia prior to metabolic profiling. While this method allowed for the standardized measurement of OXPHOS and glycolysis using Seahorse assays, the additional culture step might potentially alter cellular metabolic states and potentially mask or distort their *in situ* metabolic characteristics. Future research employing more immediate ex vivo analysis, such as the flow cytometry-based metabolic profiling (Erny et al., 2022), may provide a more actual snapshot of microglial metabolism.

Taken together, a three-month treadmill exercise intervention ameliorates AD pathology and associated cognitive deficits in APP/PS1 mice by restoring both glycolytic and OXPHOS functions in microglia, while shifting their metabolic profile from glycolytic dominance toward increased oxidative phosphorylation.

## Data Availability

The raw data supporting the conclusions of this article will be made available by the authors, without undue reservation.
