# Supplementary figures and images for: Treadmill exercise alleviates Alzheimer’s disease pathologies in APP/PS1 mice through modulation of microglial glucose metabolic reprogramming
Source: Front Aging Neurosci. 2025 Dec 19;17:1734837. doi: 10.3389/fnagi.2025.1734837 (PMC12757337; doi:10.3389/fnagi.2025.1734837)

**WT-SED**

**AD-SED**

**AD-EXE**

**AD-EXE**

**WT-SED**

**AD-EXE**

**AD-SED**

**AD-SED**

**WT-SED**


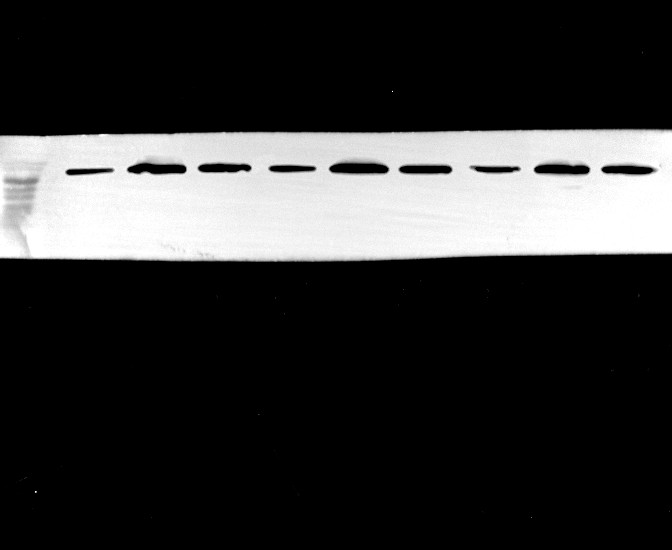
 NLRP3


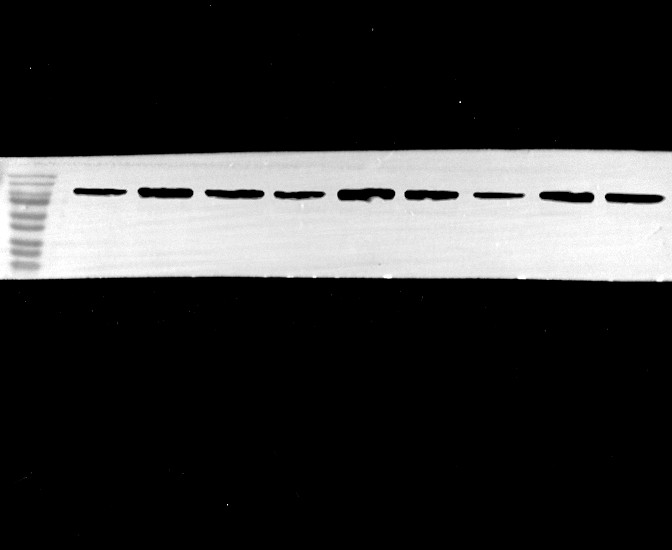


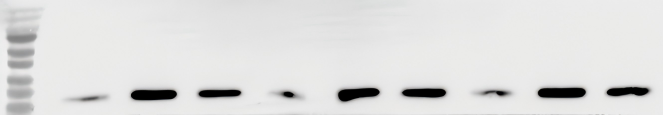
ASC


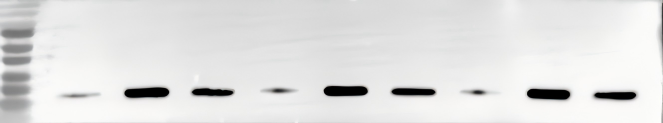


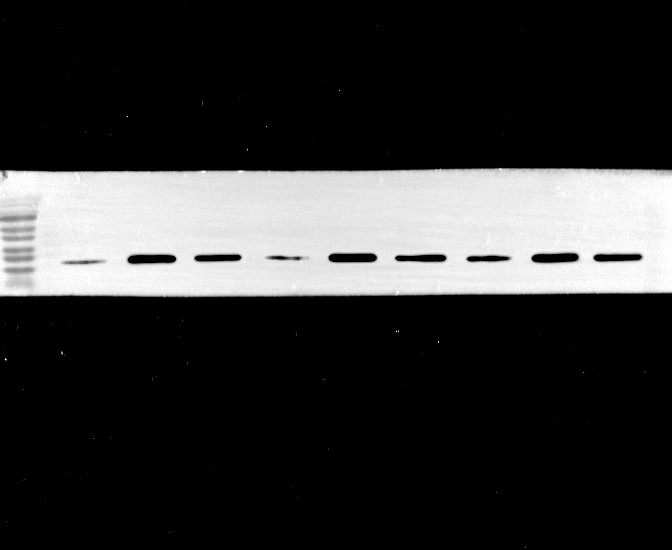
caspase-1 p20


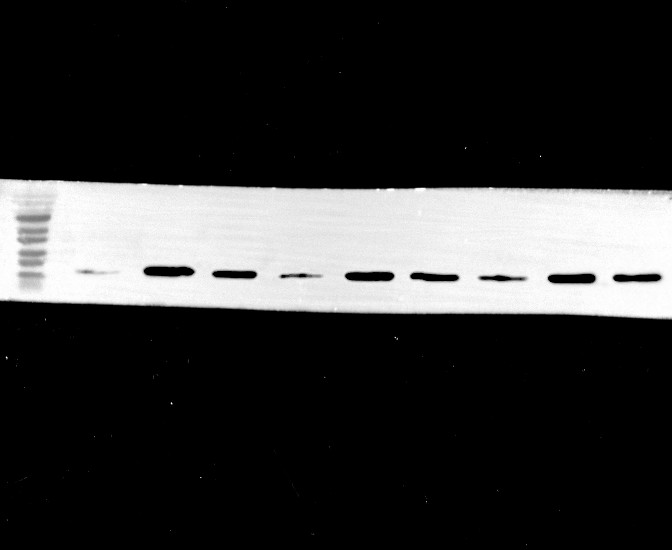


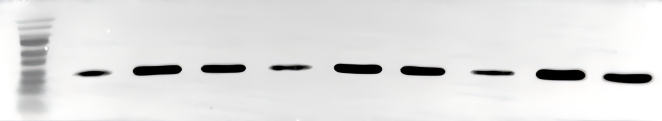
IL-1β


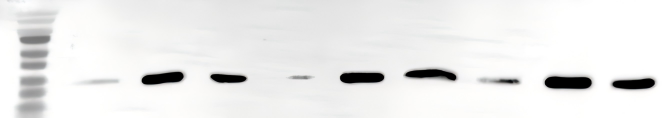


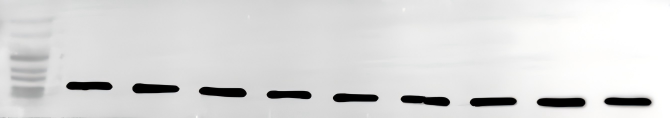
GAPDH


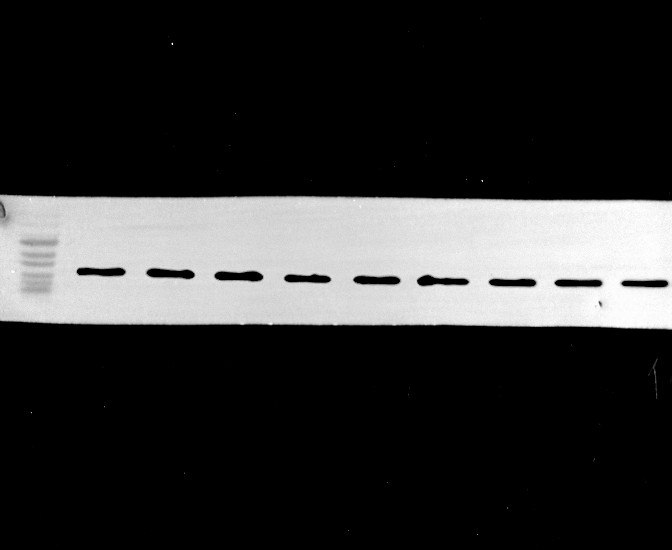

Supplement: Supplementary file 1 [file Table_1.doc]
